# Wasting and stunting are still prevalent in children with sickle cell anaemia in Lagos, Nigeria

**DOI:** 10.1186/s13052-016-0257-4

**Published:** 2016-05-04

**Authors:** Christopher I. Esezobor, Patricia Akintan, Adebola Akinsulie, Edamisan Temiye, Titilope Adeyemo

**Affiliations:** Department of Paediatrics, College of Medicine, University of Lagos, P.M.B 12003 Lagos, Nigeria; Department of Paediatrics, Lagos University Teaching Hospital, Lagos, Nigeria; Department of Haematology and Blood Transfusion, College of Medicine, University of Lagos, Lagos, Nigeria; Department of Haematology and Blood Transfusion, Lagos University Teaching Hospital, Lagos, Nigeria

**Keywords:** Obesity, Overweight, Sickle cell anaemia, Stunting, Wasting

## Abstract

**Background:**

Sickle cell anaemia (SCA) is associated with growth failure. However, recent reports indicate high rates of overweight or obesity among children with SCA in developed countries. It is unclear whether overweight or obesity is also common in children with SCA in developing countries. The objectives of the study were to determine the prevalence of overweight or obesity, wasting and stunting and identify predictors of wasting and stunting among children with SCA in Nigeria.

**Method:**

Children with SCA attending a public-funded tertiary hospital clinic were studied. Weight, height, haemoglobin, haemoglobin fractions and white cell count were measured. Anthropometric values were converted to z scores and referenced to the WHO Child Growth Standards and WHO Reference 2007. The proportions with wasting, stunting and overweight or obesity were determined. Regression analysis was used to identify the predictors of wasting and stunting.

**Results:**

Two hundred and thirty-three children [mean (±SD) age of 9.0 (±4.0) years, 60.9 % males] participated in the study. Wasting, stunting and overweight or obesity rates were 22.7 %, 11.6 % and 1.7 %, respectively. Boys and children from low socioeconomic class were 3.25 (1.45-7.29) and 2.42 (1.14-5.18) times more likely to be wasted respectively, while both wasting and stunting were more common with increasing age [adjusted OR of 1.33 (1.18-1.51) and 1.15 (1.01-1.32) respectively]. Sickle cell-related complications and intake of oral penicillin and hydroxyurea were not associated with wasting and stunting.

**Conclusion:**

Overweight or obesity is uncommon while wasting and stunting are still prevalent in children with SCA in Lagos. The strongest predictors of wasting and stunting were older age, male gender and low socioeconomic status.

## Background

Sickle cell disease (SCD) is traditionally associated with growth failure and delayed puberty [1, 2]. Among the SCD genotypes, children with sickle cell anaemia (SCA) and HB Sβ^0^ thalassemia have the most severe impairment in growth [2, 3]. Similar to the general population, growth failure in SCD has been associated with adverse events including higher hospitalisation rate [4]. With improved care children with SCD are living longer compared to life expectancy several decades ago [5, 6]. The improvement in the survival rate and overall health of children with SCD has been attributed in part to early diagnosis of SCD, penicillin prophylaxis, immunisation, stroke prevention, administration of hydroxyurea and chronic blood transfusion [5–8]. Reports from developing countries indicate that these measures are also being increasingly used in the population with SCD [9, 10]. Together, these measures have resulted in better growth of children with SCD. Indeed, recent studies from developed countries like the USA indicate that overweight and obesity, together with its complications such as hypertension and sleep apnoea are becoming a health concern among children and adolescents with SCD [11, 12]. One small study involving children less than six years with SCD in Nigeria, a developing country, has reported a similar pattern of overweight and obesity, in keeping with the trend in developed countries [13]. In contrast, another small study in Lagos involving children and adolescents with SCA reported a 2.5 % prevalence rate of obesity [14].

Although recent reports from developed countries indicate that overweight and obesity are becoming prevalent in the population with SCD it is unclear whether this trend is emerging or established in children with SCD in developing regions of the world. In the present study we determined the prevalence of wasting, overweight or obesity and stunting among children and adolescents with SCA and identified factors including demographic, sickle cell-related complications and interventions that may be associated with wasting and stunting.

## Methods

The study was conducted in the Paediatric Haematology Clinic of the Lagos University Teaching Hospital between October 2014 and September 2015. It is a part of a larger study aimed at determining the prevalence and genetic risk of sickle cell nephropathy. All consecutive children with SCA aged between 2 and 17 years were included. Children were excluded if they were acutely ill with fever or vaso-occlusive crisis or needed immediate hospitalisation for other therapeutic purposes, had other chronic illnesses not related to SCD or had received blood transfusion in the three months preceding enrolment in the study. These children were excluded in order to have a study sample with hematologic parameters as close as possible to baseline values. The medical chart of each child was reviewed and the parents questioned about the age at diagnosis, history of sickle cell-related complications such as stroke, chronic leg ulcers, avascular necrosis of the hip, priapism, kidney disease and acute chest syndrome. The number of times each child had been hospitalised in the prior 12 months for any illness or bone pains was also documented. Other information of interests included the receipt of blood transfusion ever and in the prior 12 months and the daily use of oral penicillin and hydroxyurea.

Each child was examined with specific emphasis on height and weight. The weight and height were measured by one of two investigators using a seca^™^ weighing scale. The weight was measured to the nearest 50 g with the child wearing light clothing while the height was measured to the nearest 0.1 cm using a seca™ Leicester floor mounted stadiometer. For height measurement the child stood barefooted, erect with the occiput, shoulder blades, buttocks, calf and heel making contact with the stadiometer and the head in the Frankfurt plane. The seca^™^ weighing scale was calibrated each study day with known weights. Three millilitre of blood was obtained aseptically from each child and analysed in the laboratory for complete blood count (using automated haematology analyser) and haemoglobin fractions using automated D-10™ Bio-rad high performance liquid chromatography system. Only children with sickle cell anaemia were included in this analysis because of the small proportions with other sickle cell syndromes. Socioeconomic status of the family of each child was determined using the method described by Olusanya et al. [15] which utilises the father’s occupation and mother’s highest education.

### Statement of ethics and informed consent

The study obtained ethical approval from the Lagos University Teaching Hospital’s Health Research Ethics Committee before the commencement of the study. In addition all caregivers of children included in the study provided written informed consent. Assent was also obtained from children 7 years or older.

### Data analysis

Analysis of the study data was performed using IBM SPSS Statistics version 21 (IBM Corp, Armonk, New York, USA). In addition anthropometric variables of each child were converted to z scores and referenced to the WHO Child Growth Standards and WHO Reference 2007 using the WHO Anthroplus software [16]. Weight for age z score was not computed for children 10 years or older because the WHO Reference 2007 did not provide standards for this age group. Using this reference, children with height for age z (HAZ) score < -3, <-2 to -3, -2 to +3 and > +3 were classified as severe stunting, stunting, normal and very tall, respectively, while BMI for age z (BMI z) score < -3,-3 to < -2, -2 to +2, > + 2 to +3 and > +3 referred to severe wasting, wasting, normal, overweight and obese, respectively [17].

Univariate and multivariate analyses for factors associated with wasting and stunting were performed including factors that have been reported in the literature to be associated with nutritional status in the general and sickle cell population. The factors included simultaneously as independent variables in the multiple logistic models were age, age at diagnosis of SCA, gender, socioeconomic status, presence of sickle cell-related complications, hospitalisation in the previous year, haemoglobin level, percent foetal haemoglobin, white cell count and use of oral penicillin and hydroxyurea. Categorical variables such as gender, use of oral penicillin, obesity, wasting were presented as proportions. Normally distributed data were presented as mean (±standard deviations) while skewed data were summarised as median (range). All analysis was two-tailed and *p* value <0.05 was considered statistically significant.

## Results

Two hundred and thirty-three (233) children with SCA were included in the study. The mean age was 9.0 years and 60.9 % were males. In the previous one year before the study 33.5 % (*n* = 78) were hospitalised at least once for some illnesses and 23.1 % (*n* = 54) for bone pain. Except for acute chest syndromes, small proportions (<3 % each) of the study participants had experienced sickle cell-related complications such as stroke, leg ulcers or avascular necrosis of the hip. About a quarter of the children were receiving hydroxyurea and oral penicillin each. All the nine children on chronic transfusion received blood transfusions for only six months, the last transfusion being at least three months before participation in the study; they were also receiving hydroxyurea. About half had never received a blood transfusion (Table 1). The mean HAZ and BMI z scores were -0.59 and -1.13 respectively.

**Table 1:** Characteristics of the children with sickle cell anaemia studied

	*n* = 233
Socio-demographic	
Male, *n* %	142 (60.9)
Age, mean (SD), years	9.0 (4.0)
Adolescents (10-17 years), *n* %	98 (42.1)
Socio-economic class of family	
High, *n* (%)	3 (1.3)
Middle, *n* (%)	138 (59.2)
Low, *n* (%)	92 (39.5)
Severity of sickle cell anaemia	
SCD diagnosis at or before age 12 months, *n* %	101 (43.3)
Previous year hospitalisation	
0	155 (66.5)
1	55 (23.6)
2	12 (5.2)
>2	11 (4.7)
Severe bone pain previous year^a^	
0	179 (76.8)
1	39 (16.7)
2	8 (3.4)
>2	7 (3.0)
Blood transfusion ever, *n* (%)	118 (50.6)
Blood transfusion previous year, *n* (%)	41 (17.6)
Acute chest syndrome, *n* (%)	33 (14.2)
Hip avascular necrosis, *n* (%)	6 (2.6)
Leg ulcers, *n* (%)	6 (2.6)
Stroke, *n* (%)	5 (2.1)
Priapism, *n* (%)	14 (9.9)^b^
Haemoglobin level, mean (SD), g/dL	8.4 (1.2)
Foetal haemoglobin, median (range), %	8.0 (1.0-28.6)
Interventions (current receipt)	
Chronic transfusion, *n* (%)	9 (3.9)^c^
Hydroxyurea, *n* (%)	59 (25.3)
Daily oral penicillin, *n* (%)	52 (22.3)
Anthropometry	
Weight, kg	25.8 (10.3)
Height, m	1.28 (0.21)
Body mass index, kg/m^2^	14.8 (2.07)
Height for age z score	−0.59 (1.32)
Body mass index z score	−1.13 (1.30)

^a^severe bone pain crisis was one requiring hospitalisation for more than 24 hours

^b^percentage of only male children; ^c^these children last received blood transfusion at least 3 months before study participation; these children were also taking hydroxyurea

**Fig. 1:**
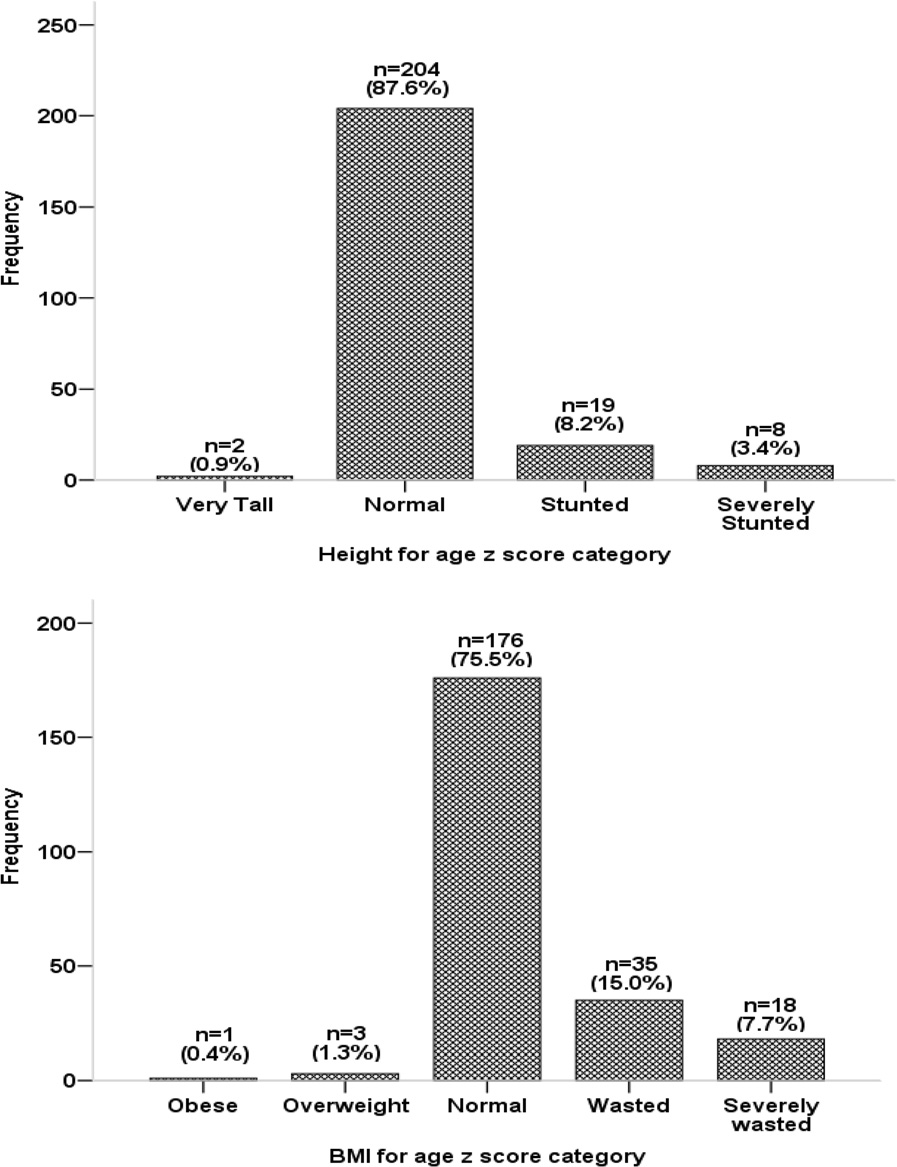
Nutritional status of children with sickle cell anaemia

Of the children studied, 27 (11.6 %) were either stunted or severely stunted while 75.5 % children had normal HAZ score. Wasting/severe wasting was observed in 22.7 % of the sample studied; 1.7 % children were either overweighed or obese (Fig. 1).

### Factors associated with wasting

**Fig. 2:**
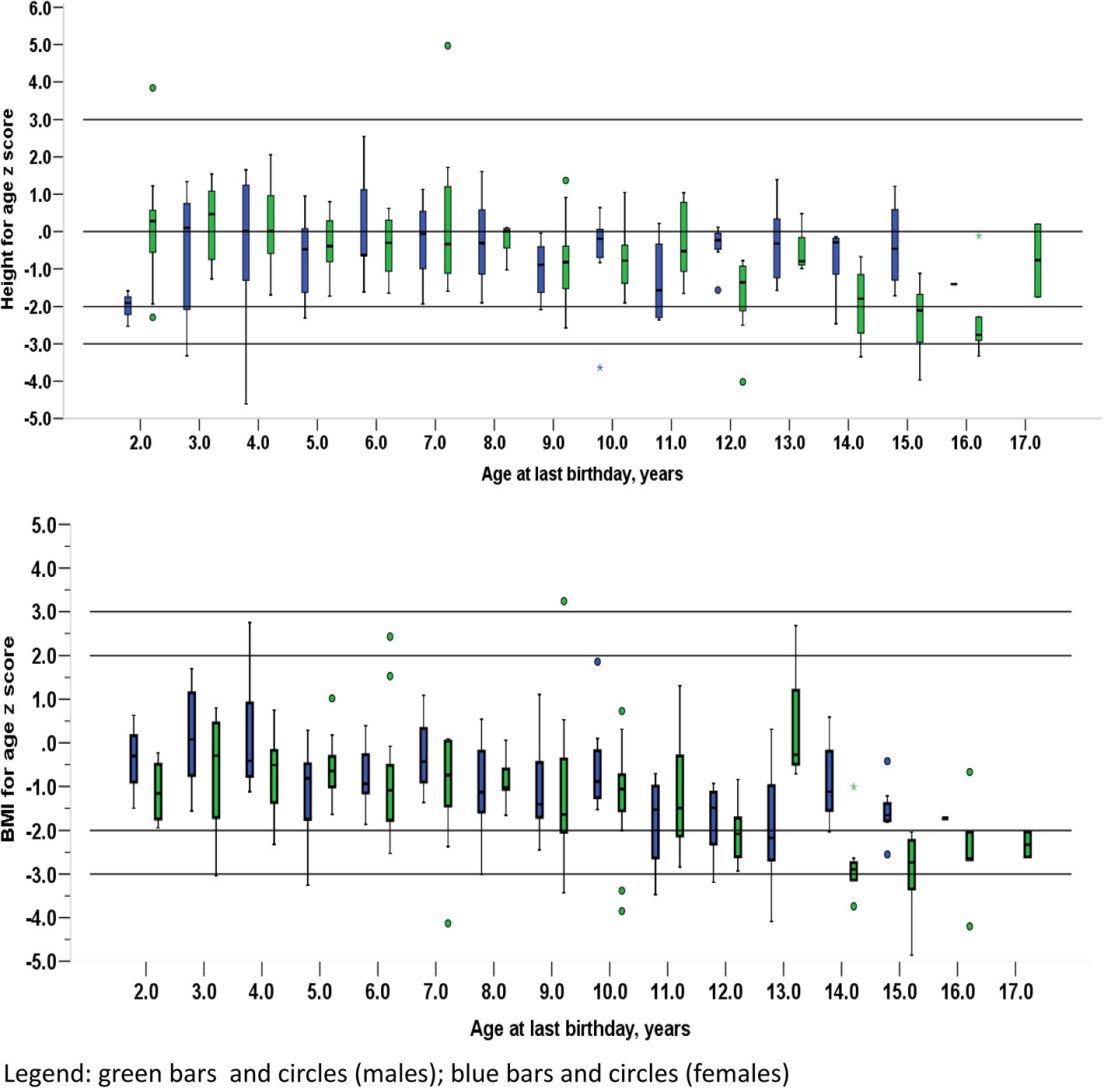
Age and gender-related decline in BMI for age and height for age z scores in children with sickle cell anaemia.

Children with wasting/severe wasting were shorter than children with normal weight (-1.31 versus -0.38). In addition, the demographics of the children with wasting/severe wasting were different from those with normal weight; they were older, predominantly males (75.5 %) and more likely to come from families with low socioeconomic status than children with normal weight. They were also more likely to be diagnosed with SCA at a later age than children with normal weight (Table 2 and Fig. 2).

**Table 2:** Factors associated with wasting and stunting in children with sickle cell anaemia

Characteristics	Wasting			Stunting		
	Wasted/severely wasted, *n* = 53	Normal weight *n* = 180	*P* value	Stunted/Severely stunted, *n* = 27	Normal Height *n* = 206	*P* value
Nutritional status						
Weight, mean (SD), kg	28.5 (8.9)	25.0 (10.5)	0.03	24.9 (9.4)	25.9 (10.4)	0.632
Height, mean (SD), m	1.41 (0.17)	1.24 (0.21)	<0.01	1.27 (0.25)	1.28 (0.20)	0.752
BMI, mean (SD), kg/m^2^	13.89 (1.41)	15.50 (2.09)	<0.001	15.00 (1.71)	15.15 (2.11)	0.700
Weight for age z score^a^, mean (SD)	−1.98 (0.87)	−0.51 (1.04)	<0.001	−2.04 (1.10)	−0.58 (1.06)	<0.001
Height for age z score, mean (SD)	−1.31 (1.23)	−0.38 (1.28)	<0.001	−2.79 (0.68)	−0.30 (1.10)	<0.001
BMI for age z score, mean (SD)	−2.82 (0.65)	−0.63 (0.98)	<0.001	−1.86 (1.61)	−1.03 (1.22)	0.002
Demographics						
Age, mean (SD), year	12.2 (3.7)	8.1 (3.6)	<0.001	11.2 (5.0)	8.7 (3.8)	0.002
Adolescent, *n* (%)	38 (71.7)	60 (33.3)	<0.001	18 (66.7)	80 (38.8)	0.006
Age at diagnosis of SCA, median(range) year	2.00 (0-14.0)	1.8 (0-12.0)	0.046	2.0 (0.0-12.0)	2.0 (0.0-14.0)	0.310
Male, *n* (%)	40 (75.5)	102 (56.7)	0.014	16 (59.3)	126 (61.2)	0.849
Low socioeconomic status, *n* (%)	31 (58.5)	61 (33.9)	0.001	13 (48.1)	79 (38.3)	0.327
SCA-related features						
≥1 SCA-related complication^b^, *n* (%)	15 (28.3)	39 (21.7)	0.314	6 (22.2)	48 (23.3)	0.901
Severe bone pain, previous year, *n* (%)	12 (22.6)	42 (23.2)	0.916	5 (18.5)	49 (23.8)	0.542
Hospitalisations, previous year, *n* (%)	16 (30.2)	62 (34.4)	0.564	8 (29.6)	70 (34.0)	0.652
Blood transfusion, ever, *n* (%)	33 (62.3)	85 (47.2)	0.054	15 (55.6)	103 (50.0)	0.587
Haemoglobin level, mean (SD), g/dL	8.3 (1.6)	8.4 (1.1)	0.927	8.4 (2.1)	8.4 (1.1)	0.764
Foetal haemoglobin level, (range), %	6.4 (1.4-19.1)	8.5 (1.0-28.6)	0.035	5.7 (1.3-15.8)	8.2 (1.0-28.6)	0.014
Total white cell count, x10^3^/ml	12.9 (4.2)	13.8 (4.7)	0.212	15.2 (6.5)	13.4 (4.3)	0.051
SCA-related interventions						
Daily oral penicillin intake, *n* (%)	4 (7.5)	48 (26.7)	0.003	3 (11.1)	49 (23.8)	0.137
Hydroxyurea or chronic transfusion, *n* (%)	9 (17.0)	50 (27.8)	0.112	3 (11.1)	56 (27.2)	0.071

^a^weight for age z score presented for 135 children younger than 10 years ^b^any of leg ulcer, acute chest syndrome, stroke, avascular necrosis of the hip, priapism or kidney disease

However, markers of severity of sickle cell anaemia such as the presence of one or more SCA-related complications such as acute chest syndrome, leg ulcers, stroke, priapism and avascular necrosis of the hip were similar in both. Also, the hospitalisation rate in the previous one year before the study and the haemoglobin level were similar. In contrast, children with wasting had significantly lower foetal haemoglobin percent than children with normal weight (6.4 % versus 8.5 %). While the proportion receiving hydroxyurea or chronic blood transfusion was not different, significantly fewer children with wasting were receiving daily oral penicillin (7.5 % v 26.7 %).

### Factors associated with stunting

In addition to being stunted children with stunting had lower BMI z score than those with normal height. Similar to wasting children with stunting were older than children with normal HAZ score (11.2 versus 8.7 years) and had lower foetal haemoglobin percent (5.7 % versus 8.2 %). However, no other demographic variables or features related to sickle cell anaemia were associated with stunting, though children with stunting had higher white cell count and were less likely to be receiving hydroxyurea.

In multiple logistic regressions (Table 3), only age was a consistent predictor of both wasting and stunting in the present study; with increasing age the likelihood of wasting and stunting was higher (adjusted OR of 1.33 and 1.15, respectively). The strongest predictor of wasting was the male gender (adjusted OR of 3.25) followed by lower socioeconomic status (adjusted OR 2.42). On the other hand stunting was independently predicted by age and peripheral blood white cell count. Other markers of severity of SCA or interventions such as the use of oral penicillin or hydroxyurea were not significantly associated with either stunting or wasting.

**Table 3:** Independent predictors of wasting and stunting in children with sickle cell anaemia

Predictors	Wasting (BMI z score < -2)		Stunting (HAZ score < -2)	
	Adjusted odds ratio (95 % CI)	*P* value	Adjusted odds (95 % CI)	*P* value
Age	1.33 (1.18–1.51)	<0.001	1.15 (1.01–1.32)	0.036
Male (versus female)	3.25 (1.45–7.29)	0.004	0.80 (0.33–1.97)	0.629
Low SES^a^ (versus middle & high)	2.42 (1.14–5.18)	0.022	1.23 (0.50–3.03)	0.653
Age at diagnosis of SCA	1.00 (0.87–1.15)	0.990	1.12 (0.954–1.31)	0.167
Presence of ≥1 SCA–related complication	0.81 (0.33–1.96)	0.638	0.70 (0.24–2.08)	0.525
Previous year hospitalisation	0.76 (0.33–1.74)	0.514	0.70 (0.26–1.89)	0.586
Haemoglobin level	1.01 (0.75–1.35)	0.968	1.21 (0.87–1.67)	0.258
Foetal haemoglobin	0.99 (0.90–1.08)	0.767	0.91 (0.80–1.03)	0.116
Peripheral white cell count	0.98 (0.90–1.08)	0.680	1.16 (1.04–1.28)	0.007
Daily oral penicillin use	0.61 (0.18–2.07)	0.423	0.78 (0.19–3.16)	0.723
Hydroxyurea or chronic transfusion	0.54 (0.21–1.37)	0.197	0.50 (0.13–1.86)	0.300

^a^ *SES* socioeconomic status

## Discussion

The present study was premised on the increasing prevalence of overweight and obesity in the general population of children globally and newer reports describing similar trend in children with sickle cell disease, a condition previously associated with significant growth failure. In the present study, we observed that wasting and stunting are still prevalent among children with SCA in a developing country like Nigeria, while overweight and obesity are uncommon. Other recent studies from developing countries have reported similar findings. For example, in 2012, Cox et al. [4] in Tanzania reported that 15 % of a cohort of children and adults with SCA had wasting or severe wasting while a higher percentage had stunting. Although a recent study in south east Nigeria reported higher rates of overweight and obesity, suggesting that overweight and obesity might be prevalent among children with SCD in developing countries, reclassifying the study sample using standard BMI z score of >2 and >3 for overweight and obesity respectively resulted in only 3.4 % of the study population being overweighed or obese [13]. Another recent study in Lagos reported an obesity rate of 2.5 % among 1-15 year old with SCA [14]. Together with the current study, these reports indicate that overweight and obesity are uncommon among children with SCD in developing countries such as Nigeria. Undernutrition rates higher than the findings of the present study have also been documented in children with SCA within the past decade. For example, Al-Saqladi [18] observed that 35 % and 54 % of a cohort of children with SCD less than 15 years were wasted and stunted respectively. The high prevalence of wasting and stunting in the present study despite increasing coverage of SCD-related health maintenance therapies is worrisome. In addition, the low percentages of children in the present study who had experienced sickle-related complications and the low hospitalisation rates for bone pain crisis or any illness in the previous one year would suggest a not too severe SCA phenotype, yet wasting and stunting were common.

In sharp contrast, reports from developed countries paint an increasing problem of overweight and obesity among the population with SCD. Recently, *Chawla* et al. [11] observed overweight or obesity in over 20 % of children with SCD in the USA. In that study the percentage of children with underweight was a third of the overweight or obesity rate. Another study in the USA reported similar findings; among 6-18 year olds with SCD 16-26 % had overweight or obesity using Centre for Disease Control reference values [12]. The increasing prevalence of overweight and obesity, two conditions with significant adverse health impact, among children with SCD in developed countries has mirrored the availability of effective SCD-related interventions in these regions of the world. Specifically, children with SCD in developed countries are more likely to be diagnosed in early infancy and started on pneumococcal prophylaxis, hydroxyurea and chronic transfusion than children in developing countries like Nigeria [5, 9]. These interventions have been associated with fewer infections, stroke, hospitalisation rates and higher haemoglobin level [6–8]. For instance the steady state haemoglobin reported in the study by Chawla [11] in the US was 9.5 g/dL compared with 8.4 g/dL in the present study and 7.3–8.3 g/dL in a study in Yemen which reported higher rate of wasting and stunting [18]. As observed in many studies, higher haemoglobin is associated with better nutritional status [11, 19, 20]. The higher prevalence of undernutrition in the studies from developing countries especially those in Africa may also be due to inclusion of only those with SCA anaemia in the studies. Compared to the other haemoglobin genotypes SCA is the most severe form of SCD [2, 11].

Both stunting and wasting occur together in children with sickle disease underlining the chronicity of SCD and the frequent exacerbations of symptoms. In the present study children with wasting were shorter than children with normal weight while those with stunting were more likely to be wasted than children with normal height. In addition, consistent with other studies, weight was more affected than height [21, 22]. Stunting and wasting in SCD is attributed to higher resting energy expenditure [23], repeated infections [24], endocrine dysfunction [25], micronutrient deficiency [26] and chronic anaemia [4, 11, 19]. The chronic nature of SCD means that after birth and with increasing age stunting and wasting become more common. Our finding supports this pattern of undernutrition as both stunting and wasting were more common among the adolescents and got severe with increasing age. Indeed only age was a strong predictor of both wasting and stunting in the present study. This association has been reported by several other investigators in both developing and developed countries and in studies involving both children and adults [4, 11]. It has been shown that with increasing age energy intake becomes inadequate in children with SCD [27].

The present study and published literature identify the male children with SCA as the subgroup most affected by growth impairment. In the present study the males were three times more likely to be wasted than girls. Chawla [11] and Mitchell [12] in the USA and Cox [4] in Tanzania separately documented that underweight or wasting was more frequent among males than females. The higher resting energy expenditure in boys compared to girls and the increased likelihood of males to be involved in outdoor physical activities may explain this finding [28]. In addition, higher background inflammation and erythroid hyperactivity have been suggested as other reasons for the higher rate of undernutrition in male children with SCD [18]. The consistency of this observation in several studies identifies males with SCD as a group requiring additional nutritional intervention such as increased daily calorie intake. Furthermore, measures such as chronic blood transfusion may have beneficial effect as they lower erythroid hyperactivity and resting energy requirement [29].

Not unexpectedly, in the present study and others children belonging to families with lower socioeconomic class were more likely to be wasted [19, 30]. Higher socioeconomic class implies better access to healthcare and better nutrition, more so in developing countries where healthcare spending is usually out of pocket. For example, Wolf et al. [19] observed that children with SCD whose family head had higher education had higher weight and height, while Zemel et al. [31] documented higher BMI z scores in girls whose mothers had high educational level. It is therefore conceivable that the rate of wasting and stunting may be higher in children with SCA in rural regions of Nigeria than the values obtained in the present study.

Our study suggests that in low-resource countries nutritional status of children with SCA is still heavily influenced by variables such as age, gender and socioeconomic status and not necessarily by the factors related to severity of SCA or its management. We did not find any association between nutritional status and markers of severity of SCA such as sickle-related complications, hospitalisation and bone pain rates in the previous year and haemoglobin level. Similarly, sickle cell-related interventions such as daily oral penicillin and hydroxyurea were only weakly protective against wasting and stunting in the present study. Although several studies have observed lower haemoglobin level in children with SCD and poorer nutritional status the association with nutritional status has not been consistently reported in the literature [11, 31, 32]. For example a study involving children in Yemen did not find any association between nutritional status and markers of severity of SCA [18]. Similarly, the impact of hydroxyurea therapy on growth status has not been consistent [33, 34]. Specific to the present study, the weak association between use of hydroxyurea and nutritional status may be due to the practice in the study centre to commence hydroxyurea only in children with or at risk of severe manifestations such as stroke and priapism. The association between stunting and higher peripheral blood white cell count has been previously reported [33]. It is thought that the higher white cell count may underline the ongoing chronic inflammation in sickle cell disease which may impair growth [33]. However, we do not know any reason why it was only associated with stunting but not wasting in the present study.

### Limitations

A major limitation of the present study was the reliance on self-reports for hospitalisation rates and most complications of sickle cell disease. This is partly because children with chronic disease such SCD in developing countries also receive care in smaller, usually private hospitals and these events are not adequately captured in the medical charts in the tertiary hospital. Another limitation was our inability to comment on the effect of chronic blood transfusion on nutritional status in the present study because only very few children included in this study received the therapy. Although higher white cell count in the blood suggests inflammation, apart from excluding children with febrile illness or acute complications of SCD we did not measure other sensitive markers of inflammation such as C reactive protein, erythrocyte sedimentation rate and serum transferrin

## Conclusion

In a cohort of children with SCA attending an urban public-funded tertiary hospital in Lagos, Nigeria, overweight or obesity was uncommon. Instead about 1 in 10 and 1 in 5 children with SCA had stunting and wasting respectively. Stunting and wasting were more common and severe in males and adolescents. However, sickle-cell related factors such as hospitalisation rates, history of complications and use of oral penicillin and hydroxyurea were not associated with wasting or stunting in these children.

## Abbreviation

BMI z score: Body mass index for age z score
HAZ score: Height for age z score
SCA: Sickle cell anaemia
SCD: Sickle cell disease
SD: Standard deviation
